# Double-blind randomized placebo-controlled multicenter clinical trial (phase IIa) on diindolylmethane’s efficacy and safety in the treatment of CIN: implications for cervical cancer prevention

**DOI:** 10.1186/s13167-015-0048-9

**Published:** 2015-12-21

**Authors:** Levon Ashrafian, Gennady Sukhikh, Vsevolod Kiselev, Mikhail Paltsev, Vadim Drukh, Igor Kuznetsov, Ekaterina Muyzhnek, Inna Apolikhina, Evgeniya Andrianova

**Affiliations:** Russian Scientific Center of Roentgenoradiology of the Ministry of Health of the Russian Federation, Moscow, 86, Profsouznaya Str., Moscow, 117837 Russia; Federal State Budget Institution “Research Center for Obstetrics, Gynecology and Perinatology” of the Ministry of Health of Russian Federation, 4, Akademika Oparina Str., Moscow, 117198 Russia; Federal State Autonomous Educational Institution of Higher Education “Peoples’ Friendship University of Russia” (PFUR), 6, Mikluho-Maklaya Str., Moscow, 117198 Russia; Federal State Budget Institution «Russian Academy of Sciences», 14 Leninsky av., Moscow, 119991 Russia; State Budgetary Educational Institution of Higher Professional Education “Moscow State Medical Stomatological University named after A.I. Evdokimov” (MSMSU) of the Ministry of Health of Russian Federation, 20 Delegatskaya Str., Build. 1, Moscow, 127473 Russia; CJSC “MiraxBioPharma”, 12, Kutuzovsky av., Build. 2, Moscow, 121248 Russia; CJSC “IlmixGroup”, 12, Kutuzovsky av., Build. 2, Moscow, 121248 Russia

**Keywords:** Cervical intraepithelial neoplasia, CIN I, CIN II, Diindolylmethane, Clinical trial, Targeted prevention, Predictive preventive personalized medicine

## Abstract

**Background:**

The article presents the results of a clinical trial on the efficacy and safety of a novel pharmaceutical composition in the form of vaginal suppositories containing diindolylmethane in the course of cervical intraepithelial neoplasia (CIN) I–II conservative treatment. It offers an attractive drug therapy for more personalized prevention of cervical cancer.

**Methods:**

A total of 78 women of reproductive age were included. This was a multicenter, randomized, placebo-controlled, double-blind, parallel-group trial with efficacy determined by histological evaluation of cervical biopsies. The efficacy of active drug treatment (100 and 200 mg/day) in both treatment groups was significantly higher in comparison with the placebo group, according to the primary efficacy end point (proportion of patients with complete CIN regression after 90–180 days of the study drug treatment).

**Results:**

The efficacies were 100.0 % (confidence interval (CI) 95 %: 82.35–100.00 %), 90.5 % (CI 95 %: 69.62–98.83 %), and 61.1 % (CI 95 %: 35.75–82.70 %), for the high dose, low does, and placebo, respectively. Adverse events in the placebo group were reported in 22 % of patients (CI 95 %: 7.5–43.7 %); in the first treatment group (100 mg/day), adverse events were reported in 40.0 % of patients (CI 95 %: 21.1–61.3 %); in the second treatment group (200 mg/day), adverse events were reported in 42.0 % of patients (CI 95 %: 22.1–63.4 %). The differences in side effects between treatment groups treated with the active drug and placebo were statistically significant. No serious adverse events were reported in any of the groups.

**Conclusions:**

Thus, the use of diindolylmethane in the form of intravaginal suppositories can be effective in patients with CIN I–II and is not accompanied by clinically significant side effects. This approach could be a better option for young women with CIN I–II as it takes in attention their reproductive plans.

**Trial registration:**

ID: ChiCTR-INR-15007497 (2 December 2015)

## Background

Cervical intraepithelial neoplasia (CIN) represents dysplastic changes in the cervical epithelium which tend to develop over time and can lead to cervical cancer (CC) [1]. There are 528,000 new cases of cervical cancer across the world every year, half of which are fatal [2]. So, it is obvious that public health needs to improve its cervical cancer prevention strategy. According to the WHO classification (1995), there are three degrees of CIN: mild (CIN I), moderate (CIN II), and severe (CIN III) dysplasia. Any CIN stage is treated based on contemporary standards, but these standards could contain a more personalized approach. This paper is focused on the attempt to make cervical cancer prevention both more preventive and more personalized.

It is known that the human papillomavirus (HPV) infection is a key causative factor of pathological changes in the cervix leading to its neoplastic transformation. Integration of the viral DNA into the cellular genome leads to an increased unregulated synthesis of viral E6 and E7 oncoproteins, which increases the risk of malignant transformation of the cervical epithelium [3, 4]. DNA of high-risk HPV types 16 and 18 is found in 50–80 % of samples of the squamous cervical epithelium with signs of moderate to severe dysplasia [5]. HPV has the potential to trigger the irreversible process of carcinogenesis of the cervical epithelium in cases where the immune system of an infected woman is weakened. CIN I resolves spontaneously in 50–60 % of cases within 3 years. At the same time, intraepithelial neoplasia develops within 2 years in 15–30 % of women infected with high-risk HPV [6], while about 10–20 % of CIN III cases transform into invasive cervical cancer [7].

Recently, there has been increasing evidence that the oncogenic activity of E6 and E7 oncoproteins can, in addition to the well-known mechanism, be realized through interaction between the p53 and retinoblastoma pRb proteins, as well as via epigenetic mechanisms. As it is known, DNA methylation and histone deacetylation causing epigenetic “silencing” of the tumor-suppressor genes are the main methods of the epigenetic regulation of gene activity [8]. Methylation is carried out using a special enzyme—DNA methyltransferase (DNMT)—or, rather, a family of three isoenzymes—DNMT1, DNMT3a, and DNMT3b. The degree of histone acetylation is regulated by special enzymes—acetylases (histone acetyltransferase (HAT)) and deacetylases (histone deacetylase (HDAC)). Numerous epigenetic changes were identified at all stages of carcinogenesis of the cervix. It has been shown that the level of methylation of tumor-suppressor genes increases with the progression of CIN [9, 10]. According to other reports, the E6 oncoprotein activates the expression of DNA methyltransferase DNMT1 and suppresses the expression of the p53 tumor-suppressor protein in cervical epithelial cells infected with HPV type 16 [11]. It has been found that the E7 oncoprotein is also able to modulate the DNA methylation mechanism. In a 2006 study [12], it was demonstrated that the E7 protein of HPV type 16 binds to DNMT1 in vitro and in vivo, stimulating the activity of the enzyme. The E7 oncoprotein is able to bind histone to deacetylase directly and recruit the enzyme to promoters of tumor-suppressor genes. There is evidence that this may lead particularly to the depression of IFN-beta gene transcription, followed by the inhibition of interferon production [13]. The E7 oncoprotein’s binding to HDAC reduces its effect on the promoter of the E2F2 transcription factor, causing an increase in the latter’s expression, leading to disruption of the cell cycle and uncontrolled cell proliferation [13].

The hormonal (estrogen) factor is another well-known pathogenetic factor in the development of CIN. In the course of metabolic conversions, the cytochrome P450 enzymatic system produces hydroxyestradiol, which is oxidized to estrone, followed by hydroxylation with the formation of two main metabolites: 16α-hydroxyestrone (16α-OHE1) and 2-hydroxyestrone (2-OHE1). It has been shown that 16α-OHE1 binds firmly to the estrogen receptor (ER) that induces hyperproliferation of epithelial cells. An alternative pathway of estrogen metabolism is 2-hydroxylation. It is associated with a reduced risk of developing hormone-dependent tumors. The ratio of metabolites 2-OHE1/16α-OHE1 ≥ 2:1 is considered optimal to maintain the normal estrogen balance [14]. It is known that the proliferation of HPV-infected cervical epithelial cells is enhanced by increased levels of 16α-OHE1 [15].

The most common treatment for CIN is a fast local destruction or excision of the affected tissue, such as loop electrosurgical excision procedure (LEEP) of the cervix, cold-knife cone biopsy, laser ablation, or cryotherapy. These methods have proved their efficiency. However, there is a risk of damaging the cervix when using them. Beyond this, cervical excision has a negative effect on pregnancy outcomes (ectopic pregnancies and terminations, miscarriage in the second trimester), especially in those patients who are repeatedly exposed to such procedures [16]. In addition, after such treatment, there is a high rate of recurrence of the disease [17]. Along with local destructive methods of treatment, in some cases, patients undergo immune stimulation therapy with interferon. However, this approach does not lead to stable clinical results in the majority of cases [18]. Due to this, it seems appropriate to develop optimal schemes of systemic and local pharmacological correction using targeted agents acting on the molecular mechanisms of CIN pathogenesis and subsequent malignant transformation.

Indole-3-carbinol (I3C) and its stable metabolite 3,3′-diindolylmethane (DIM) are substances with proven multitarget activity affecting a wide range of molecular mechanisms. They can significantly slow down, stop, or even regress neoplastic processes in the cervical epithelium [19, 20].

It has been shown that I3C and DIM have a specific anti-estrogen effect by stimulating processes of estradiol 2-hydroxylation under the influence of cytochrome P450, thereby increasing the overall concentration of 2-OHE1, a physiological metabolite relative to 16α-OHE1, an “aggressive” metabolite [21]. It was experimentally confirmed that I3C and DIM inhibited the growth of estrogen-sensitive tumors in vivo and displayed antitumor activity in CIN patients [22–26]. DIM causes apoptosis and arrests the growth of virus-infected cervical cancer cells in vitro and in vivo [27, 28]. Another important feature of DIM is its ability to inhibit tumor stem cells selectively. According to recent studies, these are the main cause of tumor recurrence and metastases [29]. The epigenetic anticancer effect of DIM has been proven. DIM causes DNA-demethylating activity and has the ability to inhibit the activity of class I histone deacetylases, restoring the activity of tumor-suppressor genes [30, 31]. Also, previously detected DIM immunomodulating activity contributes to the potential reversibility of the pathological changes to the cervix [23]. Therefore, DIM is active against multiple HPV-infected cervical epithelial cells. The development of topical DIM-based medications for CIN treatment is relevant due to the possibility of achieving higher concentrations of the active substance in the affected tissue, as well as reducing the likelihood of adverse reactions due to gastrointestinal tract exposure.

The purpose of this study was to investigate the efficacy and safety of a new pharmaceutical formulation of DIM used in the form of vaginal suppositories for treatment of women diagnosed with CIN I–II. Despite CIN I being rarely treated, we wanted to check whether a safe and effective therapy for CIN I could be developed. The main reason was that a possibly cheap and safe therapy may become a common option for these patients in contrast to surgical treatment.

## Methods

The study included 78 patients of reproductive age with abnormal colposcopy findings and histological verification of CIN I and/or CIN II. Common inclusion criteria for all the patients were the following: written informed consent to participate in this study; diagnosis of grades 1–2 cervical intraepithelial neoplasia (CIN I–II), histologically verified; age of 18–39 years; ability to carry out the procedures according to the trial protocol; no official or other forms of relations to the persons involved in the study interested in its outcomes; and consent to use a barrier method of contraception (condoms) during the whole period of the study. The barrier contraception method was recommended in order to minimize concurrent genitourinary infection and to decrease the bias.

Exclusion criteria were the following: pregnancy or breast-feeding, severe cervical intraepithelial neoplasia (CIN III) or cancer in situ, malignant tumors of any localization, infections of the genitourinary system in the acute phase, diseases of the cardiovascular and nervous system and/or concomitant renal or hepatic failure, positive syphilis or HIV tests, alcohol abuse and/or narcotic or drug dependency, and intake of any investigational drug within 30 days prior to first dose of the study drug.

The current clinical trial had a multicenter, randomized, placebo-controlled, double-blind design and was carried out in three parallel groups. The trial was conducted in 12 study sites located in the Russian Federation. The trial was approved by the Ministry of Health (resolution number 399 dated 10 October 2012, http://grls.rosminzdrav.ru/CIPermitionReg.aspx) and local Ethics Committees of the study sites. The study’s hypothesis on the effectiveness of the drug is that its use should lead to a more frequent recovery from CIN compared to a placebo. Recovery was defined as complete elimination of CIN according to histological studies of cervical biopsies. Thus, if the rate of recovery from CIN by the end of the study in the placebo group and in each of the two study groups is equal to h0, h1, and h2, respectively, null and alternative hypotheses can be formulated as follows:

- For the comparison of placebo and DIM therapy in the dose of 100 mg/day:
- H01: h0 = h1, there are no treatment-dependеnt differences in the incidence of CIN recovery.
- H11: h0 ≠ h1, there are treatment-dependеnt differences in the incidence of CIN recovery.
- For the comparison of placebo and DIM therapy in the dose of 200 mg/day:
- H02: h0 = h2, there are no treatment-dependеnt differences in the incidence of CIN recovery.
- H12: h0 ≠ h2, there are treatment-dependеnt differences in the incidence of CIN recovery.

The need to verify the hypothesis of the study was used as a basis in order to perform calculations of the proper sample size. Statistical power was taken as 80 % (*β* = 0.2), and the significance level (*α*) was taken as 0.05 (two-tailed). As we were going to perform two pairwise Holm-adjusted comparisons [32], the sample size was calculated according to the lowest *α*/2 level = 0.025. It was decided that the hypothesized effectiveness of treatment in each of the study groups may reach 85 %; for the purposes of calculating the effectiveness in the placebo group, this was taken as 45 % (on the basis of the published data on the incidence of spontaneous CIN regression) [33–35]. For *β* = 0.20 and *α* = 0.025, with the assumed difference between the rates of the two study groups being *p*_2_ − *p*_1_ = 0.40, we needed 26 subjects with available data for analysis in each group.

Calculation of the sample size was performed as follows:

$$ n={\left[{Z}_{\alpha }+{Z}_{\beta}\right]}^2\ast \left[\left({p}_1\ast \left(1-{p}_1\right)\right)+\left({p}_2\ast \left(1-{p}_2\right)\right)\right]/{\left[{p}_1-{p}_2\right]}^2, $$

where *n*—sample size for each group; *p*_1_—rate in the first group; *p*_2_—rate in the second group; *p*_2_ − *p*_1_—difference between rates of both groups; and *Z*_*α*_ and *Z*_*β*_—critical values of distribution corresponding to a given level of error types 1 and 2, respectively.

A randomization list was provided by the Sponsor before the beginning of the study using SPSS Statistics version 20.0.0 computer software. Block randomization was used with a block size equal to 3; each block containing two patients who were assigned different doses of the active drug and one patient who was assigned a placebo. The (blinded) treatment assignment procedure was carried out by sending a fax to the Sponsor from the study site. The fax contained a randomization request form with information on the patient’s conformity with the inclusion/exclusion criteria. The Sponsor’s response, sent by fax to the study site, consisted of a patient report form with a unique identification number and drug identification code on the packaging (in accordance with the randomization sheet, packages of the preparation contained an appropriate daily dose of the active drug or a placebo). The randomization sheet was kept solely by the Sponsor.

Forty-five days before the start of the study, the patients were screened: a process including the collection of medical history, physical examination, clinical and biochemical blood tests, urinalysis, electrocardiogram, pelvic ultrasonography, colposcopy with photography, and cervical biopsy. After clarification of the criteria for inclusion and non-inclusion and the decision on whom to include in the study, patients were randomized into three groups: women from group 1 (*n* = 26) received an intravaginal form of DIM in the dose of 200 mg/day (one suppository of the study drug twice a day (BD)), women from group 2 (*n* = 26) received an intravaginal form of DIM in the dose of 100 mg/day or a placebo (one suppository of the study drug and one suppository of placebo per day), and women from group 3 (control group (*n* = 26)) received only the placebo intravaginally (one suppository with placebo BD). The course of treatment lasted 180 days without a break (therapy was terminated in patients who had complete CIN regression according to histological examination on day 90 of the study). Investigators and patients did not have any information on whether the preparation being taken was the active drug or a placebo. The study drug was “Cervikon-DIM” (CJSC “IlmixGroup,” Russia) in the form of a vaginal suppository. Each suppository was composed of the active substance (3,3′-diindolylmethane 100 mg) and excipients (solid fat 1.9795 g, crospovidone 0.02 g, carmine red dye 0.0005 g). The placebo suppository consisted of solid fat 2.0795 g, crospovidone 0.02 g, and carmine red dye 0.0005 g. The placebo was indistinguishable from the active drug by its packaging, appearance, and organoleptic characteristics.

The study program included four visits: a baseline visit (visit 1), then visits after 30 days (visit 2), 90 days (visit 3), and 180 days (visit 4) from the beginning of the therapy. All women underwent a comprehensive survey during all visits, including clinical blood and urine analyses, electrocardiogram, cytology, and colposcopy with photography.

The treatment efficacy was assessed by the proportion of patients with complete CIN regression on the basis of the histological examination of biopsy specimens of the cervical lesions—the “gold standard” in CIN diagnosis. Histological examination of biopsies of cervical lesions was performed at the screening and after 90 and 180 days of therapy. Withdrawal of biological samples for morphological evaluation was carried out with colposcopic visualization using special biopsy forceps for conchotomy in order to avoid coagulation of the biopsy’s edges. A biopsy and histological study was performed on all the patients after 90 days of therapy. In the absence of CIN, the patient’s intake of the study drug was terminated. Indicators as to whether patients who discontinued treatment due to CIN recovery on day 90 should have a biopsy after 180 days were the identification of an abnormal transformation zone, aceto-white epithelium with mosaic and/or punctate, iodine-negative epithelium, abnormal blood vessels, or other potentially CIN-associated features during the colposcopy after 180 days. If the colposcopy (on day 180) was normal, a biopsy was not performed in these patients 180 days after therapy began, and it was assumed that they had made a full recovery from CIN. With patients who took the study drug throughout the 180 days, a biopsy was carried out in all cases at the end of the study.

Histological examination was performed sequentially by two blinded independent laboratories: first of all by a local one at the study site and then in a central laboratory. Where there were discrepancies in the results, the final decision was taken by the central laboratory after reading the opinion of the local laboratory. At the end of the study, we counted the number of patients in whom the absence of CIN was confirmed according to the morphological study. The persistence of CIN foci in biopsies was considered a negative response to the treatment.

The safety of the treatment was evaluated by detecting adverse events (AEs). In addition, we assessed the parameters of vital body functions, the data from objective studies and lab parameters at each patient’s visit: body weight, body mass index, systolic blood pressure, diastolic blood pressure, heart rate, respiratory rate, body temperature, urinalysis (transparency, color, specific gravity, pH, protein, glucose, ketone bodies, erythrocytes, leukocytes, cylinders, salts, microorganisms), common blood count (hemoglobin, red blood cell (RBC), white blood cell (WBC), erythrocyte sedimentation rate), biochemical blood analysis (total protein, creatinine, glucose, alanine transaminase (ALT (GPT)), aspartate transaminase (AST (GOT)), total bilirubin), and electrocardiogram (ECG). Deviations in laboratory parameters and ECG were defined by the investigator as AEs if they demanded, in his opinion, further diagnostic and/or therapeutic measures.

We used a Kruskal-Wallis test to compare the homogeneity of the sample in the groups. A Clopper-Pearson method was used for calculating 95 % binomial confidence intervals. A mixed-designed ANOVA model for the analysis of variance with repeated measures was used to analyze the relationship between vital function variations and instrumental/laboratory data, treatment group, and visit (change in time). To compare the frequencies between the groups, we used Fisher’s exact test. For planned comparisons of the efficacy, we calculated two-sided significance. The assessment of significance was performed following the stepwise procedure of the Bonferroni correction modified by Holm [32]. This procedure involves carrying out tests with the order of significance decreasing. The lower of two *p* values is tested at the level of *α*/2. If the corresponding hypothesis is rejected, then the second *p* value is tested at the level of *α*; otherwise, both null hypotheses are accepted. The total level of significance for this test procedure is equal to *α*. Statistical analysis of the results of the study was performed using SPSS Statistics software, version 20.0.0 (IBM Corporation).

## Results

The women enrolled in the study were aged from 19 to 39. The average age of patients in group 1 was 26 years; in groups 2 and 3, it was 27 years. Thus, CIN I–II occurred most commonly in young women. This confirms the results of numerous studies suggesting that CIN is more prevalent in younger women than in older women. Study participant statistics are shown in Table 1.

**Table 1:** Distribution of the subjects into groups and the study population

Intervention	Group			Total
	DIM	DIM	Placebo	
	100 mg/day	200 mg/day		
Number of patients enrolled	26	26	26	78
Date of inclusion of the first patient	10 December 2012	10 December 2012	21 December 2012	
Date of inclusion of the last patient	21 November 2013	20 November 2013	20 November 2013	
Date of the last visit of the last patient	4 April 2014	5 April 2014	15 April 2014	
Eliminated from the study due to withdrawal of consent to participate in the study prior to receiving the first dose of the drug and excluded from the analysis (both safety and efficacy)	1	2	3	6
Eliminated from the study due to withdrawal of consent to participate in the study after the beginning of therapy	2	4	4	10
Ceased to meet the criteria for participation in the study	0	0	0	0
Excluded from the study for safety reasons ahead of schedule	2	1	1	4
Completed visit procedure in 90 days of treatment in accordance with the study protocol and included in the efficacy analysis	22	20	19	61
Stopped taking the drug due to CIN recovery after 90 days of therapy	15	17	10	42
Completed visit procedures after 180 days of treatment in accordance with the study protocol and included in the efficacy analysis	21	19	18	58

*CIN* cervical intraepithelial neoplasia, *DIM* 3,3′-diindolylmethane

The selected groups were virtually homogeneous in terms of baseline comparative values at the time of screening. The demographic characteristics of the study groups (for each group) and the total sample analysis are presented below (Tables 2 and 3). When preparing summary tables in this section, we did not include patients who were withdrawn prior to initiating the therapy.

**Table 2:** Baseline demographic characteristics of the study groups and in the total sample of the study (e.g., race, smoking, history of the disease, comorbidities)

Index		Group						Total	
		DIM		DIM		Placebo			
		100 mg/day		200 mg/day					
		Rate	% in group	Rate	% in group	Rate	% in group	Rate	% of all
Race	Caucasian	24	100	23	100	23	100	70	100
	Other	0	0	0	0	0	0	0	0
Smoking	No	23	92	24	100	23	92	70	94.6
	Yes	2	8	0	0	2	8	4	5.4
Significant diseases in anamnesis	No	20	87.0	18	78.3	19	82.6	57	82.6
	Yes	3	13.0	5	21.7	4	17.4	12	17.4
The presence of comorbidities	No	18	78.3	17	74.0	20	87.0	55	79.7
	Yes	5	21.7	6	26.0	3	13.0	14	20.3

*DIM* 3,3′-diindolylmethane

**Table 3:** Baseline demographic characteristics and vital signs in the study groups and in the overall analytic sample (mean ± standard deviation)

Parameter	Group			Total
	DIM	DIM	Placebo	
	100 mg/day	200 mg/day		
Age, years	27 ± 5	27 ± 3	27 ± 5	27 ± 5
Height, cm	167 ± 6	166 ± 7	165 ± 5	166 ± 5
Body weight, kg	63 ± 11	59 ± 10	64 ± 13	62 ± 11
Body mass index	23 ± 4	22 ± 3	23 ± 5	23 ± 4
Systolic blood pressure, mmHg	112 ± 8	110 ± 7	111 ± 9	111 ± 8
Diastolic blood pressure, mmHg	73 ± 6	68 ± 5	71 ± 5	71 ± 6
Heart rate, beats per minute	73 ± 5	72 ± 6	72 ± 7	73 ± 6
Respiratory rate, breaths per minute	16 ± 2	17 ± 4	17 ± 4	17 ± 3
Body temperature, °C	37 ± 0	37 ± 0	37 ± 0	37 ± 0
Age of menarche, years	13 ± 1	13 ± 1	14 ± 2	13 ± 1
Duration of menstruation, days	5 ± 1	5 ± 1	5 ± 1	5 ± 1
Duration of the menstrual cycle, days	29 ± 2	29 ± 4	29 ± 2	29 ± 3

*DIM* 3,3′-diindolylmethane

The study groups had no significant differences in terms of key demographic indicators, gynecological history, and vital signs, which suggests that the study sample was homogeneous. The findings from physical examinations were normal in all the subjects.

**Fig. 1:**
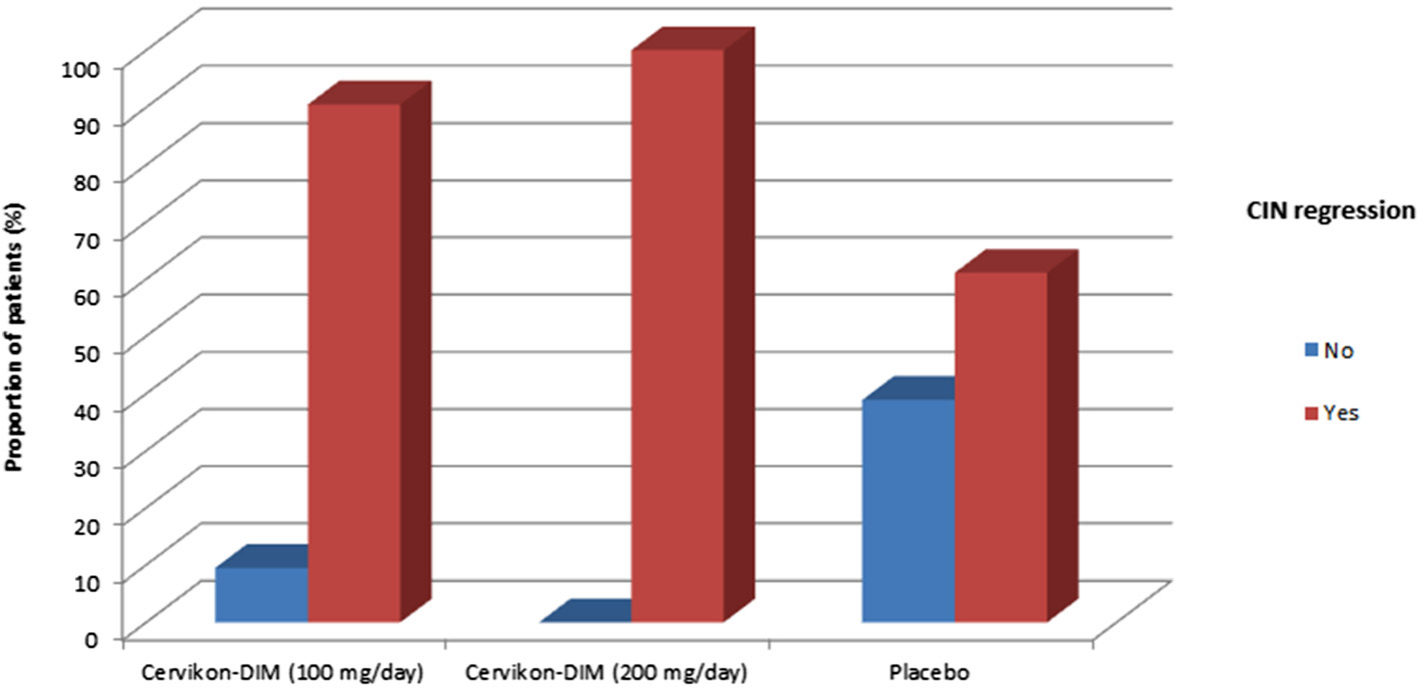
Effectiveness of the therapy according to the data of histological examination (90–180 days of treatment)

When comparing the efficacy of the therapy (CIN regression on histological examination) after 180 days, we found that in the treatment group receiving a 100 mg/day dose of DIM, the therapy efficacy was higher than that in the placebo group (Fig. 1), amounting to 90.50 % (confidence interval (CI) 95 %: 69.62–98.83 %) and 61.10 % (CI 95 %: 35.75–82.70 %), respectively. In the group receiving a 200 mg/day dose of DIM, the therapy efficacy was higher than that in the placebo group (Fig. 1), amounting to 100.0 % (CI 95 %: 82.35–100.00 %) and 61.10 % (CI 95 %: 35.75–82.70 %), respectively.

The smallest *p* value for comparing the frequency of CIN absence between groups is 0.003. Thus, taking into account the Holm correction, first of all, we tested the H02 hypothesis at the level of *α* = 0.025 (Table 4). Testing the H02 hypothesis has shown that these differences are statistically significant, and the null hypothesis is rejected. Subsequently, testing of the H01 hypothesis yielded *p* = 0.0361 (taking in account the Holm correction amended by Hill); the difference was thus statistically significant, and the null hypothesis was rejected at the pre-determined level of *α* = 0.05. Thus, the efficacy of the study therapy is considered to be statistically significantly higher than the placebo therapy in each of the experimental groups.

**Table 4:** The results of evaluation of the effectiveness of CIN therapy in the study groups (positive effect—absence of CIN)

CIN regression	Group							
	DIM 100 mg/day			DIM 200 mg/day			Placebo	
	Number of patients	% in group	*p* (compared to placebo)	Number of patients	% in group	*p* (compared to placebo)	Number of patients	% in group
No	2	9.5	0.0361	0	0	0.003	7	38.9
Yes	19	90.5		19	100		11	61.1

*CIN* cervical intraepithelial neoplasia, *DIM* 3,3′-diindolylmethane

In total, during this study, we recorded 34 AEs (13 in the treatment group taking DIM in the dose of 100 mg/day, 14 in the treatment group taking DIM in the dose of 200 mg/day, and 7 in the placebo group). We excluded patients who were withdrawn before taking the first dose of the study drug from the safety analysis. The developed undesirable phenomena were distributed relatively evenly in the patients during the whole study (Table 5).

**Table 5:** Number of patients with AEs after visits 1–4 in the study group

AEs after a visit	Group								
	DIM			DIM			Placebo		
	100 mg/day			200 mg/day					
	(*n* = 25)			(*n* = 24)			(*n* = 23)		
	Rates	Percent	95 % CI	Rates	Percent	95 % CI	Rates	Percent	95 % CI
Visit 1	0	0.0	0.0–13.7	1	4.2	0.1–21.1	3	13.0	2.8–33.6
Visit 2	7	28.0	12.1–49.4	3	12.5	2.7–32.4	1	4.3	0.1–22.0
Visit 3	2	8.0	1.0–26.0	6	25.0	9.8–46.7	1	4.3	0.1–22.0
Visit 4	3	12.0	2.6–31.2	4	16.7	4.7–37.4	2	8.7	1.1–28.0

*AE* adverse events, *CI* confidence interval, *DIM* 3,3′-diindolylmethane

In the placebo group, AEs occurred in 22 % of patients (5 of 23 subjects, CI 95 %: 7.5–43.7 %); in the group treated with a 100 mg/day dose of DIM, AEs were reported in 40.0 % of patients (10 of 25 subjects, CI 95 %: 21.1–61.3 %); and in the group treated with a 200 mg/day dose of DIM, AEs were reported in 42.0 % of patients (10 of 24 subjects, CI 95 %: 22.1–63.4 %). We found significant differences in the number of patients who developed AEs between the groups treated by the active drug as compared to the placebo group (DIM 100 mg/day, *p* = 0.0045; DIM 200 mg/day, *p* = 0.0019).

In total, we revealed 23 kinds of AEs, out of which 3 were associated with particular changes in urinalysis, 5 were associated with variations in the clinical and biochemical blood analysis, 2 were associated with ECG changes, 5 were associated with subjective sexual function disorders (discomfort in the vagina, etc.), and 8 were associated with other types of AEs (Table 6).

**Table 6:** Types of adverse events, recorded in the study groups

AEs reported in the patient (in alphabetical order)	Group						Total	
	DIM		DIM		Placebo			
	100 mg/day		200 mg/day					
	Frequency	% in group	Frequency	% in group	Frequency	% in group	Frequency	% of all
Bacteriuria, leukocyturia, proteinuria	0	0.0	1	7.1	0	0.0	1	2.9
Myocardial hypoxia on ECG	0	0.0	1	7.1	0	0.0	1	2.9
Dysbiosis of the vagina	0	0.0	1	7.1	0	0.0	1	2.9
Discomfort when using the drug	1	7.7	0	0.0	0	0.0	1	2.9
Burning, itching of the vagina	4	30.8	1	7.1	0	0.0	5	14.7
Loose stool	1	7.7	0	0.0	0	0.0	1	2.9
Delay of menstruation	0	0.0	1	7.1	0	0.0	1	2.9
Slower growth of hair on the head, body	0	0.0	1	7.1	0	0.0	1	2.9
Ketone bodies in urine	0	0.0	1	7.1	0	0.0	1	2.9
Urticaria	1	7.7	0	0.0	0	0.0	1	2.9
Leukocytosis in the cervical smear	0	0.0	1	7.1	0	0.0	1	2.9
Abundant flocculent discharge from the genital tract	0	0.0	2	14.3	1	14.3	3	8.8
Acute respiratory viral infection	1	7.7	1	7.1	3	42.9	5	14.7
Increased blood pressure	0	0.0	1	7.1	0	0.0	1	2.9
Increased plasma levels of ALT (GPT) and AST (GOT)	0	0.0	2	14.3	0	0.0	2	5.9
Increased erythrocyte sedimentation rate	1	7.7	0	0.0	0	0.0	1	2.9
Proteinuria	1	7.7	0	0.0	0	0.0	1	2.9
Decrease in hemoglobin in the blood	0	0.0	0	0.0	1	14.3	1	2.9
Decrease in plasma creatinine	1	7.7	0	0.0	0	0.0	1	2.9
Reduced white blood cell count	0	0.0	0	0.0	1	14.3	1	2.9
Dryness in the vagina during sexual intercourse	1	7.7	0	0.0	0	0.0	1	2.9
Increased amount of white vaginal discharge	0	0.0	0	0.0	1	14.3	1	2.9
Shortening of the AVC	1	7.7	0	0.0	0	0.0	1	2.9
Total	13		14		7		34	

*AE* adverse events, *ALT (GPT)* alanine transaminase, *AST (GOT)* aspartate transaminase, AVC (atrioventricular conduction) , *DIM* 3,3′-diindolylmethane, *ECG* electrocardiogram

In 4 cases out of the 34 AEs, the researcher at the local site considered that the AEs had developed as a result of the therapy by the study drug (burning, itching of the vagina—2 cases; urticaria—1 case; acute respiratory viral infection—1 case). Among other AEs, in 5 cases, there was a probable relationship between the study drug intake and AE development from the researcher’s perspective; in 5 cases, the relationship was possible; in 10 cases—doubtful; in 1 case—conditional; and in 9 cases, the investigator was unable to classify the relationship with AEs. In 18 cases out of 34, no AE therapy was performed; in 16 cases, specialized treatment was conducted. The severity of AEs was marked as “moderate” for one case of acute respiratory viral infection; in one case, there was an increase in the amount of white vaginal discharge; in one case, there was myocardial hypoxia on the electrocardiogram; there were two cases of burning and itching in the vagina; and in one case, researches found profuse, flaky discharge from the genital tract. In all other 28 cases, the severity of the AE was marked as “mild.” In four cases, the study was terminated prematurely due to AEs: one case (burning and itching in the vagina) in the treatment group taking DIM in the dose of 200 mg/day, two cases (burning and itching in the vagina, urticaria) in the treatment group taking DIM in the dose of 100 mg/day, and one case (acute respiratory viral infection) in the placebo group.

No serious AEs were recorded in this study.

In the vast majority of cases, there were no statistically significant changes in these indicators during the therapy, and no relationship with the study drug was revealed, according to the analysis of parameters of vital body functions, the data from objective medical examinations, and instrumental/laboratory parameters in the study groups by visit dynamics. A relationship has been identified regarding systolic and diastolic blood pressure (we found the impact of the group effect to be *p* = 0.021 and *p* = 0.010, respectively; the values were lower in the treatment group taking a 200 mg/day dose of DIM) and in the plasma levels of ALT and AST (it was shown that there is an impact of the group effect: *p* = 0.034 and *p* = 0.018, respectively; the values were lower in the treatment group taking a 200 mg/day dose of DIM).

## Discussion

Currently, there is no single approach to the treatment of CIN I and/or CIN II in young women. The existing standards justify an aggressive radicalism, which often leads to the disruption of reproductive function. CIN is most prevalent in younger women. We have confirmed this thesis in this study as well. Destructive methods of CIN treatment are invasive and traumatic, despite their proven efficacy. They often disrupt the patient’s reproductive plans. At the same time, waiting for long-term follow-up to check for spontaneous regression of CIN and the tactics of non-interference (especially when the patient cancels visits to the doctor) can permit further progression of the disease process. Therefore, searching for an adequate conservative treatment is an important issue in the management of CIN I–II. This approach could be very valuable and is in accordance with the EPMA (European Association for Predictive, Preventive and Personalized Medicine)  position in the cancer prevention field [36]. A number of randomized, placebo-controlled clinical trials have reviewed in detail the relationship between the use of oral I3C and DIM substances in CIN patients and noted an improvement in the course of the disease during treatment with these substances.

The study of Bell et al. involved 17 women diagnosed with CIN II–III [20]. For 12 weeks, each of them took I3C per os in a dose of 200 or 400 mg per day. At the end of the study, there was complete CIN regression in four of the eight patients who received a 200 mg/day dose of I3C and in four of the nine patients taking a 400 mg/day dose of I3C. At the same time, in all the women with complete CIN regression, there was a significant increase in urinary excretion of the estradiol 2-OHE1 metabolite.

In a clinical study performed by Del Priore et al., it was shown that the use of DIM per os in the dose of 2 mg/kg/day caused a clinical improvement in CIN II–III patients. However, no statistically significant differences between the experimental and control groups were revealed [37]. Probably, this result occurred due to the low bioavailability of the test DIM formulation. The data from another clinical study supports this explanation: researches tested high doses of formulated BioResponse-DIM (a crystalline diindolylmethane in a microfine starch whose bioavailability is 1.5–2 times higher than that of non-formulated DIM). At the same time, complete CIN II and CIN III recovery was observed [38].

Results of a big DIM clinical trial in CIN treatment were reported by Castañon et al. in 2012 [39]. As treatment intervention, 150 mg of oral DIM formulation or placebo (one time in a day) was used, and no statistically significant difference between groups was detected. But the serious disadvantage of this study was that baseline CIN was not histologically proven; beyond it, half-life of DIM formulation (3 h) might be too small for one time in a day administration. Our general suggestion is that a relatively higher DIM concentration is necessary for CIN treatment, and so, topical DIM administration may be preferable.

In addition, experimental diindolylmethane studies in vitro and in vivo provide a reasonable basis for its clinical evaluation as a means of prevention and treatment of cervical cancer [22, 23, 28, 40–42]. So, we can assume that a drug based on an active DIM substance should display high activity against transformed cells of the cervical epithelium.

Our study showed that the intravaginal use of DIM in doses of 200 and 100 mg/day for 90–180 days was accompanied by a high rate of CIN I–II reversion. Undoubtedly, this non-invasive method is very promising for the conservative treatment of CIN in women of reproductive age, demonstrating a careful and sparing approach which does not involve damage to the cervix. Also, this method has been proved pathogenetically, given the mechanism of DIM’s effect on cell transformation following HPV infection and the production of the E6 and E7 proteins. Local application of DIM, unlike the systemic use, is usually characterized by a higher level of safety and tolerability. The studied form of vaginal suppositories for treating CIN I–II may be considered the most safe and effective method of treatment, with minimal and rare side effects. This also supports patient compliance, taking into account the location of the affected area and topical use of the drug.

Nevertheless, this study has some limitations. Particularly, it involved a small number of patients. In addition, the data obtained can only be considered preliminary in relation to the selection of the preferred mode of treatment (daily dose and duration of treatment). Another general limitation of our trial design is that the investigator could have missed cervical lesions during colposcopy on day 180 and thus had not taken the biopsy. If this took place, it could bias the results.

Previously, a phase I clinical trial of the drug was performed (the data were not published). We successively included four groups of 10 volunteers who used an intravaginal form of the drug in an increasing dosage (in doses of 50 mg once a day (QD), 100 mg QD, 200 mg QD, and 100 mg four times a day (QID) with intervals of 0.5 h). In the study, we observed 14 AEs in 10 volunteers. Among the AEs were the following: increased erythrocyte sedimentation rate (seven cases), decreased erythrocyte sedimentation rate (one case), increase in the level of total bilirubin (one case), and increased ALT and AST (one case). No serious AEs were observed. In all cases, the researcher estimated the intensity of AEs as “minor.” The study did not reveal any significant effect of the study drug on vital signs, ECG, clinical and biochemical blood tests, and urinalysis.

In the current phase IIa study, we have found significant differences in the treatment groups taking DIM as compared with the placebo group by the number of patients who developed AEs (DIM 100 mg/day, *p* = 0.0045; DIM 200 mg/day, *p* = 0.0019). Of the 34 cases of AEs identified in this study, in 4 cases, the researchers claimed they were related to the received drug (burning and itching in the vagina—2 cases; urticaria—1 case; acute respiratory viral infection—1 case). Nevertheless, a drug effect leading to the development of respiratory viral infections is improbable, because of the lack of data on potential mechanisms of this phenomenon. In the study groups, there were no significant changes in laboratory parameters during the treatment (or they were not clinically significant in case of blood pressure) and there was no relationship with the study drug with the exception of ALT and AST. The study drug has shown the dose-related group effect (*p* = 0.034 and *p* = 0.018, respectively, for the groups of patients taking 100 and 200 mg/day doses) on the level of these transaminases. Increased AST and ALT levels in a number of patients have been recognized by some researchers to be clinically significant and reported as AEs. However, it should be noted that in virtually all cases this increase was transient. Only two patients had elevated levels of transaminases at the time of visit 4 (with an increased level of only one transaminase). Itching and burning in the vagina was noted in the patients treated with placebo and active suppositories; therefore, the response of the mucosal epithelium to excipients used in suppositories, as well as individual sensitivity, cannot be excluded. However, in order to clarify the presence of hepatotoxicity and the local irritating action of the drug, we need to perform further studies with a larger number of patients. In addition, the small number of patients receiving the study drug overall may have limited our ability to identify relatively rare side effects of the drug. Also, a higher rate of local AEs may have led to an “unblinding” effect if the investigator might have guessed that the patient was on active treatment. It may be another limitation of our trial too. Therefore, further clinical studies are needed to detect the efficacy and safety of the drug.

## Conclusions

The presented results of the safety and efficiacy of the Cervikon-DIM, obtained within the Phase IIa of the double-blind, randomized, placebo-controlled multicenter study, confirm efficacy and favorable safety profile of the investigated drug. Thus, the use of diindolylmethane in the form of intravaginal suppositories can be effective in patients with CIN I-II, and is not accompanied by clinically significant side effects.

### Expert recommendations

Drug development based on diindolylmethane’s ability to prevent cancer promotion is a technically complicated, but very promising, issue. As oral medications are preferable for prevention of most cancer localization, in some cases, topical formulations may be more appropriate. Using this substance in a suppository form could be useful for CIN treatment, especially in young women. As it is stated in the EPMA white paper [43], cervical histopathology is useful as a predictive biomarker for cervical cancer. So, a prevention strategy in young patients may be based on CIN conservative treatment with a topical drug containing diindolylmethane.

## Abbreviations

16α-OHE1: 16α-hydroxyestrone
2-OHE1: 2-hydroxyestrone
AEs: Adverse events
ALT (GPT): Alanine transaminase
AST (GOT): Aspartate transaminase
BD: Twice a day
CC: Cervical cancer
CIs: Confidence intervals
CIN: Cervical intraepithelial neoplasia
DIM: 3,3′-Diindolylmethane
DNMT: DNA methyltransferase
ECG: Electrocardiogram
ER: Estrogen receptor
HAT: Histone acetyltransferase
HDAC: Histone deacetylase
HPV: Human papillomavirus
I3C: Indole-3-carbinol
LEEP: Loop electrosurgical excision procedure
QD: One a day
QID: Four times a day
RBCs: Red blood cells
WBCs: White blood cells
